# “I need to take care of myself”: a qualitative study on coping strategies, support and health promotion for social workers serving refugees and homeless individuals

**DOI:** 10.1186/s12995-020-00270-3

**Published:** 2020-06-26

**Authors:** Janika Mette, Tanja Wirth, Albert Nienhaus, Volker Harth, Stefanie Mache

**Affiliations:** 1 Institute for Occupational and Maritime Medicine, University Medical Centre Hamburg-Eppendorf, Seewartenstr. 10, 20459 Hamburg, Germany; 2 Competence Centre for Epidemiology and Health Services Research for Healthcare Professionals (CVcare), University Medical Centre Hamburg-Eppendorf, Martinistr. 42, 20246 Hamburg, Germany; 3 Department of Occupational Medicine, Hazardous Substances and Public Health, Institution for Statutory Accident Insurance and Prevention in the Health and Welfare Services (BGW), Pappelallee 33/35/37, 22089 Hamburg, Germany

**Keywords:** Social work, Homeless, Refugees, Coping, Support, Workplace health promotion

## Abstract

**Background:**

Social workers provide support for various groups of clients, such as refugees and homeless people. Refugees and homeless individuals represent particularly vulnerable groups in precarious living conditions. Therefore, social workers serving these clients are likely to be confronted with extensive job demands. The aim of this study was to investigate the coping strategies of social workers serving refugees and homeless individuals and to explore their support sources and health promotion offers at work as well as their respective needs.

**Methods:**

26 semi-structured qualitative interviews were carried out with social workers in Berlin and Hamburg and analysed according to Mayring’s qualitative content analysis.

**Results:**

The respondents reported various coping strategies to deal with their job demands which involved both problem-oriented (e.g. time management, setting boundaries, seeking support in conflict situations) and emotion-focused approaches (e.g. self-care, distance from work, leisure activities). In addition, they emphasised various sources of workplace (social) support, e.g. provided by team members, supervisors, and other institutions. However, unmet needs for support were also formulated by the workers, e.g. in terms of individual supervision and regular exchange. Furthermore, several employees did not know about any health promotion offers at their workplace and expressed a desire for structural and behavioural health promotion measures.

**Conclusions:**

In view of the diverse needs of the workers, the results can provide a basis to design needs-based health promotion interventions for staff in social work.

## Background

### Social work in the refugee and homeless aid

In recent years, a persistent upward trend in the number of refugees and homeless individuals has been observed worldwide [1, 2]. The global refugee population is constantly increasing, reaching 25.9 million by the end of 2018 [1]. In the context of the rapidly increasing migration in Europe in 2015 and 2016, a total of 745,545 people submitted asylum applications in Germany in 2016 [3]. Since then, the number of asylum applications in Germany has declined again (2019: 165,938), which was partly due to the refugee agreement between the European Union and Turkey [3].

Homelessness has also increased substantially in most countries [2]. In 2017, there were around 650,000 homeless individuals in Germany, from which the number of homeless recognised refugees was estimated at about 375,000. In fact, there is a notable overlap between the groups of refugees and homeless people; from 2007 to 2017, an increase in the number of non-German EU citizens and non-EU citizens in homeless assistance was observed [2]. The main reasons cited for the rising number of homeless people in Germany are the insufficient supply of affordable housing, the shrinking social housing stock and the consolidation of poverty [2].

Refugees and homeless individuals represent particularly vulnerable groups. They find themselves in precarious life circumstances, are often marginalised and frequently suffer from severe traumatic experiences [4–6]. Prejudices were revealed in recent surveys. For example, in 2017, 80% of German survey respondents feared a burden on the welfare state and 72% feared an increase in social conflicts due to refugee immigration [7]. Regarding homelessness, in a representative long-term study of the German population, homeless people were perceived as unpleasant (38%) and work-shy (30%) [8]. Both groups share certain similarities in terms of their precariousness (e.g., their material situation, income, social integration) and regarding their health impairment and strain (e.g. high rates of traumatisation, comorbidities between mental health disorders and substance misuse) [4, 9]. In terms of traumatic experiences, the prevalence of traumatisation among refugee clients was found to be around 40–60%, corresponding to a significantly increased risk in these clients [4]. Overall, both refugees and homeless individuals represent important clients for today’s and future social work [1, 2].

Previous research has examined the working conditions and health of various subgroups of social workers, e.g. mental health workers [10] or child welfare workers [11, 12]. However, less attention has been paid to the situation of social workers in refugee and homeless aid. Social workers who provide counselling and care services for refugees and homeless individuals are likely to face similar demands in their daily work [13], which makes it plausible to conduct research studies that address workers for both client groups simultaneously. For example, particular stress factors for social workers serving refugees and homeless persons consist of cultural and language problems, negative attitudes from public towards their work and their clients as well as high caseloads due to the increasing number of clients [13]. Moreover, a relatively high prevalence of secondary or post-traumatic stress has been revealed in social workers serving refugees (52% [14]) and homeless clients (36% [15]).

In a recent scoping review of 25 studies, evidence on the working conditions, health and coping strategies of social workers serving refugees and homeless individuals was systematically mapped for the first time [9]. The review revealed common job demands for this staff, including high workloads, the bureaucratic system, clients’ suffering, difficulties in maintaining boundaries with clients, as well as limited success concerning the clients’ progress. Job resources of value to workers were also identified, e.g. a high personal meaning of work and social support from colleagues. Overall, there was a high prevalence of mental health problems (e.g. burnout) among social workers in these areas. At the same time, they were found to show high levels of job satisfaction. The review also demonstrated methodological issues in relation to available studies and claimed for more research to examine the effectiveness of coping strategies and workplace health promotion offers for staff in social work with refugees and homeless clients [9].

Similar results regarding these topics were obtained in our interview study in which social workers in refugee and homeless aid described high emotional demands, high word loads, a lack of personnel, and overtime work as critical job demands [13]. In contrast, the joy of working with their clients and appreciation from clients, colleagues and superiors were underlined as job resources. Strain reactions in relation to their work involved perceptions of fatigue and stress (as short-term reactions) as well as sleeping problems, depression and burnout symptoms (as long-term consequences). Moreover, some respondents stated that they felt ill more frequently and reported high levels of sickness absences within their institutions [13].

Given the recent findings on the job demands and strains experienced by social workers in the refugee and homeless aid as well as the limited evidence, it is important to address the question of how these workers deal with their job demands. Precisely, what coping strategies do they use and what sources of support and health promotion offers at work help them to maintain their health and well-being?

### Coping strategies of social workers

In previous studies, social workers in refugee and homeless aid were found to use various coping strategies to deal with their job demands [5, 6, 16–18]. They consisted of accepting the boundaries of one’s sphere of influence [18] and maintaining professional boundaries with clients [5, 16, 17] and between work and private life [5, 16, 17]. Further coping strategies were to engage in hobbies (e.g. physical activity, reading, listening to music) and to have an active social life and exchange with friends, family members, and colleagues [5, 6, 16, 17]. Moreover, coping behaviours employed by staff in the homeless sector included the acknowledgement of small successes [5] and the acceptance of clients’ undesirable behaviour without taking it personally [19]. In addition, in a study with social workers serving unaccompanied asylum-seeking refugee children, the workers used both emotion-focused (e.g. positive reappraisal, distancing) and problem-focused strategies (planful problem solving) [20].

In general, evidence from coping research suggests that coping may have an impact on the link between employees’ working conditions and health [21]. This buffering effect has also been proven in the area of social work [12, 22]. For example, using active control-oriented coping behaviours which implied personal engagement (e.g. problem solving, cognitive restructuring, expressing emotions) buffered the impact of work stress on the emotional exhaustion and job satisfaction of social workers [12, 23]. Similarly, in a study with child protection workers, the use of active and engaged coping strategies (rather than avoidant coping strategies) led to a decline in depersonalisation levels and increased employees’ sense of personal accomplishment [22].

### Sources of support for social workers

Research suggests that further sources of support at work may help social workers to deal with their job demands [16, 17, 24, 25]. In general, team support has been described as a relevant job resource for social workers [16, 17]. Furthermore, several forms of supervision and training have been highlighted in their importance [16, 24]. In a study with case managers serving homeless clients, managers were offered an occupational therapy consultant who provided client assessments and treatment recommendations [25]. The results showed that case managers who used the consultations more actively showed higher levels of job satisfaction and self-efficacy.

However, disparity was found in previous studies with regard to whether staff felt adequately supported or wished for more support at work [16]. Indeed, social workers in refugee and homeless aid expressed the need for external counselling, supervision and training (e.g. on self-protection or to better understand new policies) [5, 16, 17, 24, 26]. In a study with German refugee aid workers, the workers particularly wished for training to better recognise the mental health problems of their clients and learn about suitable intervention strategies [27]. In addition, frontline homeless workers expressed a desire for more support (e.g. in the form of manuals, additional personnel and supervision), team development activities and greater recognition of their needs [15].

### Workplace health promotion for social workers

Studies in the area of social work, in particular refugee and homeless aid, have not yet focused on the topic of workplace health promotion. Therefore, it is still unclear to what extent social workers may benefit from health promotion offers. In general, meta-analyses indicate that workplace health promotion can contribute to maintaining employees’ health and well-being, e.g. with regard to their physical activity [28, 29], dietary habits [30, 31], and mental well-being [32]. Health promotion offers were also found to be associated with reduced job stress [28] and sickness absence [28, 32, 33] as well as increased work ability [32]. Moreover, they were related to economic benefits for companies in the form of a high return on investment [34, 35]. In view of the possible positive effects of workplace health promotion, it seems worthwhile to explore the availability of such offers for the target group more closely.

### Theoretical framework

To investigate the coping strategies of social workers, the concept of coping by Lazarus and Folkman was used as a theoretical framework [36, 37]. According to this model, coping is defined as cognitive and behavioural efforts made to master, tolerate or reduce external and internal demands, as well as conflicts among them [36, 37]. Coping is seen as a buffer between stressors and health outcomes [38, 39]. Before coping behaviour is initiated, a cognitive-transactional process takes place which encompasses a primary cognitive appraisal (evaluation of the situation as potentially stressful) and a secondary cognitive appraisal (assessment of available coping resources) [36, 37]. Coping strategies either aim at managing the stress-inducing problem (problem-focused) or at regulating emotions or distress caused by the problem (emotion-focused).

To examine the sources of support for the workers, we primarily referred to the concept of workplace social support [40]. Workplace social support emanates from multiple sources, such as supervisors, colleagues and the institution. A meta-analysis concluded that workplace social support includes both an individual’s belief that one is valued, appreciated and cared for, as well as the perception that one has access to helping relationships of varying quality and strength [40].

To assess the availability of health promotion offers and social workers’ respective needs, the Luxembourg Declaration on Workplace Health Promotion provided a useful framework [41]. The declaration defines workplace health promotion as “the combined efforts of employees, employers and society to improve the health and well-being of people at work”. Health promotion offers can include behavioural and structural interventions; the former aim at changing behavioural patterns of individuals or groups, while the latter refer to environmental and political interventions to influence health-related ecological, social, cultural and technical-material environments [42].

### Study aims and research questions

The aim of the study was to investigate the coping strategies of social workers in homeless and refugee aid to deal with their job demands. In addition, we aimed to explore the sources of support and health promotion offers for these workers, as well as their respective needs. To address our study objectives, we proposed the following research questions:

1. *What coping strategies do social workers in refugee and homeless aid use to deal with their job demands?*
2. *What sources of support are available to social workers in refugee and homeless aid at their workplace?*
3. *What health promotion offers are available to social workers in refugee and homeless aid, and what are their respective needs that are currently not addressed?*

## Materials and methods

### Study design

We conducted 26 semi-structured qualitative interviews with staff in social work in Berlin and Hamburg. Interviews were carried out from October to December 2017. The qualitative approach was chosen as it allowed us to gain first explorative insights into little researched topics. Since little was known about the topics for the specific target group, a qualitative investigation was most suitable to get a comprehensive and detailed understanding. A central advantage of the qualitative method is that it allows to describe complex social phenomena from the perspective of the people affected. Semi-structured interviews were especially suitable in order to approach the target group and study the topics within their natural environment [43]. The results of the qualitative study were subsequently used as a basis to design a quantitative online survey.

### Recruitment of participants

Participants were recruited from institutions in the refugee and homeless aid sector. Purposeful sampling was applied to the selection of institutions by contacting walk-in and residential facilities from various supporting organisations. Institutions were informed about the study by telephone and sent invitation emails and leaflets which were distributed within the organisations. In total, 19 institutions were contacted from which 10 agreed to participate. Employees who were interested in participation could contact the researchers confidentially and directly to make interview appointments (convenience sample). Eligibility criteria for study participation were as follows: participants had to have direct contact with refugees and/or homeless individuals at work and at least 6 months of work experience in social work. Moreover, they had to be of full age and fluent in the German language. Volunteers and employees working in administrative services without direct contact to clients were excluded from the study.

### Data collection

A semi-structured interview guideline was developed based on the empirical evidence and theoretical background. The questions of the interview guideline regarding the coping strategies, sources of support and health promotion are provided in Additional file 1. The guideline consisted of further questions, e.g. regarding social workers’ working conditions and strains, which are presented elsewhere [13]. A pretest interview was carried out with a former social worker from refugee aid. The guideline was slightly revised based on the workers’ recommendations. The interviews were conducted by two female researchers, a health scientist and a psychologist who were experienced with qualitative research and worked as researchers in occupational health psychology during the study period. Prior to data collection, participants were informed about the study aims and data confidentiality and signed a declaration of informed consent. All interviews were carried out face-to-face and took place in the workers’ institutions during their work time. The interviews were conducted in German and recorded with an audio device. They lasted from 27 to 86 min (51 min on average). The participants were able to terminate the interviews at any time. Interviews were conducted until no new topics were identified, i.e. data saturation was reached. Field notes were made immediately after each interview. No repeat interviews were carried out.

### Data analysis

The audio recordings were transcribed verbatim and subsequently anonymised. The data analysis was carried out in a deductive-inductive process according to Mayring’s qualitative content analysis [44]. Important features of this analysis include the systematic and rule-based approach and the development of a profound category system [44]. The well-validated, rule guided process applied in Mayring’s content analysis strengthens the reliability of the qualitative results. Qualitative content analysis was chosen, since this method focuses on the content (rather than on the latent meaning) of what is said [45]. Thus, we adopted a realistic position in the theory of science by focusing on the semantic content of the data [45]. The main categories were retrieved deductively on the basis of the interview guideline. Moreover, sub-categories were developed inductively in an iterative process. First, one interview was test-coded by both interviewers and compared in terms of consensus. Disagreements were thoroughly discussed until consensus was reached and the coding system was slightly revised. The other interviews were then each coded by one interviewer. Unclear coding was regularly discussed during team meetings. The software MAXQDA Analytics Pro (version 11) was used for the analysis [46]. The final coding system was summarised in a separate document in which the material was further compacted (paraphrased, generalised and reduced) in accordance with Mayring’s specifications [44]. During the analysis, the researchers’ personal involvement, preconceptions and influence on the results and interpretations were thoroughly reflected upon. In order to minimize such personal influences, special emphasis was placed on discussing results in the team and weighing up alternative paths of interpretation together to increase validity of the findings. Results were not made available to the participants before completion of the data analysis. Direct quotes from the interviewees were translated into English by a native speaker. The COREQ-Checklist was used to describe the study [47].

## Results

### Participant characteristics

In total, 17 interviewees were female and 9 were male (Table 1). They were aged between 26 and 64 years with a mean age of 42 years. The majority had a degree in social work (*n* = 16). 14 interviewees worked in homeless aid and 12 in refugee aid. Most of the participants worked full-time (*n* = 20) and had three or less years of experience in social work (*n* = 15).

**Table 1:** Participant characteristics

Characteristics	***n***	%
**Gender**		
Female	17	65.4
Male	9	34.6
**Age (years)**		
≤ 30	6	23.1
31–40	8	30.8
41–50	4	15.4
> 50	8	30.8
Mean (range): 42 years (26–64 years)		
**Professional qualification**		
Social worker, social education worker (bachelor, diploma)	16	61.5
Educator, remedial therapist	3	11.5
Educationalist (diploma)	1	3.8
Career-changer	6	23.1
**Area of work**		
Homeless aid	14	53.8
Refugee aid	12	46.2
**Type of facility**		
Walk-in counselling centre	7	26.9
Day care centre, outreach social work	4	15.4
Initial registration centre	3	11.5
**Professional experience in social work (years)**		
≤ 3	15	57.7
4–10	6	23.1
> 10	5	19.2
Mean (range): 7 years (8 months–37.5 years)		
**Working time**		
Full-time (≥36 h)	20	76.9
Part-time (< 36 h)	6	23.1

### Coping strategies

The coping strategies presented in the following were named by the participants as strategies they actually applied and perceived as helpful. The strategies were classified into problem-oriented and emotion-oriented strategies.

#### Problem-oriented strategies

Problem-oriented strategies referred to employees’ work tasks and content, to the work organisation, social relations and personal strategies (Table 2).

**Table 2:** Problem-oriented coping strategies

**Work tasks and content**	
• Reducing work tasks • Acquiring knowledge • Independent problem-solving	
**Work organisation**	
• Having good time management • Adhering to work time and breaks • Reducing work time • Prioritising • Separating work and private life • Creating change at higher levels of the system	
**Social relations**	
• Setting limits towards clients • Acting in a calm, confident and self-determined manner in conflict situations • Seeking support in challenging situations • Overcoming language barriers • Open discussions within the team in conflict situations	
**Personal strategies**	
• Psychotherapy • Using medication against insomnia and sleep disturbances	

### Work tasks and content

Some employees reported that they had actively reduced their work tasks and only accepted tasks they were responsible for and able to finish on time. Moreover, acquiring knowledge in dealing with stress was described as a coping strategy and associated with an increased sense of security and the development of a professional identity. Knowledge was, for example, acquired through training and education, reading books and having discussions with experts in the field. Moreover, independent problem-solving and solution-oriented thinking were reported as a further coping strategy at work:

*“There is a lot of collaborative thinking involved, and a lot of ‘Yes, okay, how can we solve this now? It’s hard, of course, but we’ll try to find a solution for this now too.’ And, yes, there is a lot of willingness too.” [#21, female, homeless aid].*

### Work organisation

With respect to work organisation, having good time management was an important strategy. This included scheduling enough breaks between appointments and avoiding appointments at the beginning of a working day to be able to prepare for the day. Furthermore, compliance with regular working hours, breaks and counselling times and the avoidance of overtime work were highlighted in order to recover briefly during breaks and between consultations:

*“Yes, so at the beginning I did a lot more overtime. And, well, now I’m trying to curb that a bit. (…) Including when it comes to consulting time. “[#9, female, homeless aid].*

Two interviewees mentioned that they had deliberately reduced their work time in order to decrease their workloads, which was perceived as a relief. In situations of high workload and time pressure, another strategy named by the interviewees was to prioritise tasks that needed to be done:

*“Setting priorities. You learn that with time. What can I move, what can I let go? (…) But that simply comes with experience, which comes with time.” [#23, male, homeless aid].*

Many interviewees described that setting clear boundaries between work and private life was an essential strategy to be able to switch off from work. These people explained, for example, that they did not share their private telephone numbers with clients, did not meet clients in their spare time and did not discuss work-related problems at home with their family and friends:

*“Sometimes we even make it clear, and say that today we aren’t going to talk about work, or about clients, or about anything remotely to do with social work. Simply so that you can switch off for once.” [#22, female, homeless aid].*

Two interviewees also described their efforts to create change at higher levels of the system, e.g. through discourse with responsible staff, committee work or work situation analyses at their workplaces in order to identify critical job demands.

### Social relations

Several workers reported setting limits towards clients at work as a fundamental coping strategy. This involved showing clients the limits for aid and support, pointing out clients’ personal responsibilities and encouraging them to reflect on their (sometimes unrealistic) expectations:

*“Sometimes there are these expectations: ‘You’re my support worker, you have to solve this for me or do that for me.’ And we have to tell people again and again, I can help you with this and that issue, I’m here to help you with this, but this and that you have to do yourself.” [#24, female, refugee aid].*

Employees stated that it was particularly important to set clear boundaries in cases where clients showed demanding and aggressive behaviour or disrespectful conduct towards women:

*“And I set really clear boundaries. If they come in acting in an aggressive manner and insult me, then I say: ‘You have to leave now and when you’ve calmed down, then you can come back and we can talk to each other calmly.” And then they might come back a couple of days later and have calmed down.” [#25, male, refugee aid].*

A general coping strategy in conflict situations consisted of acting self-confidently, calmly and in a self-determined manner, while showing understanding and empathy for the clients’ needs at the same time. Moreover, actively searching for support in challenging situations was another strategy stated by many interviewees; for example, asking colleagues for help, requesting additional supervision or calling the police as a last resort in cases where clients acted in an extremely violent or aggressive manner:

*“And when it’s totally unacceptable and residents won’t calm down at all, then I just call the police.” [#22, female, homeless aid].*

In addition, overcoming language barriers with clients by communicating through gestures and mimicry was reported as a strategy by one worker. Another respondent described that it was important to discuss conflicts with colleagues and superiors (e.g. bullying, gossip) directly and openly with the involved team members.

### Personal strategies

With respect to coping strategies at a personal level, one worker mentioned receiving medical treatment for insomnia and sleep disorders. Furthermore, three interviewees stated that they had started psychotherapy to be able to talk about their job strain and learn about mechanisms to better deal with their demands at work:

*“A year ago I started psychotherapy because I was just going straight to sleep when I got home from work. And I simply wasn’t doing anything else (…). It was just work and sleep, work and sleep. So that’s why I started therapy and have learned how to deal with this strain.” [#4, female, refugee aid].*

In this regard, psychotherapy sessions were described as a substitute for a lack of collegial counselling and individual supervision that was not provided by the employer:

*“In the end I sorted out psychotherapy for myself (…). I just simply got to the point where I said, ‘Okay, I have to look after myself, because what I need isn’t happening here.’” [#6, female, refugee aid].*

#### Emotion-oriented strategies

The emotion-oriented coping strategies named by the interviewees are depicted in Table 3.

**Table 3:** Emotion-oriented coping strategies

**Social relations**	
• Seeking emotional support from family and friends • Seeking emotional support from colleagues	
**Engagement in leisure activities**	
• Engaging in sports/physical activity • Meeting friends • Spending time outside in nature • Engaging in creative hobbies • Using media (TV, computer) • Childcare • Travelling	
**Acceptance and focus**	
• Withstanding negative experiences • Focusing on positive experiences	
**Self-care and mindfulness**	
• Reporting sick when feeling ill • Knowing one’s own limits • Using relaxation techniques • Taking concrete actions for self-care (nutrition, breaks)	
**Distance to work**	
• Distancing oneself from work, especially from clients‘problems • Not taking failures personally • Creating awareness regarding clients’ self-responsibility • Alcohol consumption	

### Social relations

Seeking emotional support and exchange with partners, parents and friends represented an important emotion-oriented coping strategy for many participants, especially when there were acute problems at work. In addition, a lot of the interviewees highlighted the relevance of regular exchange with their colleagues, and sometimes also with executives:

*“There you can also (…) talk about things that are bothering you at the moment or just vent. That really helps a lot.” [#2, female, homeless aid].*

### Engagement in leisure activities

Further emotion-oriented coping strategies were related to the engagement in leisure activities to seek distraction, detachment and a balance with work. Many respondents emphasised that they preferred active activities in their spare time, such as sports or physical activity (e.g. sports courses, cycling, swimming, dancing and horse riding). Spending time and pursuing activities with friends and family was often deemed helpful. Other workers reported that they preferred calm activities as a contrast to their busy working lives, e. g. yoga, qi gong, or going to the sauna:

*“Well, I can already see that I really need to relax and recover a lot in my private life, so there you really have to find an absolute counterbalance, otherwise it gets really difficult.” [#11, female, refugee aid].*

Spending time in nature and outdoors, for example going for walks with the dog or gardening, was described as a compensation for stressful and mainly sedentary work. According to several workers, it represented a good way to switch off from work:

*“I surround myself in nature a lot. I go out with my dog and even sometimes make my journey to or from work longer and use the time to go for a walk or bike ride (…). And by doing that I definitely unwind.” [#3, female, homeless aid].*

Creative hobbies were also mentioned by some respondents, e.g. making music, singing, writing, sewing or photography. Others indicated reading or using media, such as TV or the computer, to detach from work.

### Acceptance and focus

Another coping strategy mentioned by many respondents consisted of withstanding negative feelings at work (e.g. caused by criticism regarding one’s way of working). As described by the interviewees, such feelings could be reduced by cognitively distancing oneself from criticism and confidently following one’s own work tasks. Moreover, accepting situations that could not be changed was described as important, for example, with regard to the fates of clients, when clients maintained their unrealistic expectations or did not accept help:

*“Well, just by simply saying: ‘I accept the situation as it is.’ Being able to do that is also pretty difficult, as you actually have your own ideas of how things might go for people, but it often just doesn’t work out.” [#9, female, homeless aid].*

In addition, one interviewee underlined that it was helpful to concentrate on the positive sides of work and to remind oneself and one’s clients of previous positive achievements:

*“(…) that again and again you try to concentrate on the positive things and remind yourself: What went well and what have you already achieved?” [#2, female, homeless aid].*

### Self-care and mindfulness

Further coping strategies were related to the workers’ self-care. Some interviewees stressed the importance of reporting sick in the event of illness and of taking short breaks at work despite potentially negative comments from colleagues or postponed work. Various workers talked about not being put under stress, being mindful, knowing one’s limits and not working beyond them. Increasing awareness of one’s own needs was described as essential, as well as taking concrete actions for recovery at work, e.g. using relaxation techniques, having active breaks or following healthy eating:

*“You have to watch out for that, and when you realise, you have to say: ‘Okay, good, I have to look after myself a bit too.’ So simply that you keep an eye on yourself.” [#7, female, homeless aid].*

### Distance to work

Creating distance from work, especially from clients’ problems and concerns, was described as a useful coping strategy to better deal with challenges and perceived failures in the work context. This meant taking a step back from clients’ problems and “getting a big tank”:

*“I try maybe to not let everything get to me. So (…) that they are their problems, not my problems, basically.” [#1, female, homeless aid].*

It also meant not taking failures, aggression or appointment cancellations by clients personally. Helpful mechanisms were to consciously reflect and understand that failures had nothing to do with one’s professional skills, and to recall the clients’ responsibilities:

*“It’s really a question of attitude (…), that people are always independent and act independently. And, yes, that I offer support and provide guidance, but that I can’t do things for them (…).” [#21, female, homeless aid].*

### Sources of support

There were various sources of support mentioned by the interviewees, many of them relating to workplace social support, i.e. interpersonal relationships in the work context (Table 4).

**Table 4:** Sources of support

**Sources of support**	
• Support from colleagues and supervisors • Team meetings • Supervision • Training courses • Support from other institutions • Support in private life	

#### Support from colleagues and supervisors

Social workers repeatedly stated that support was provided by their colleagues in the form of collegial advice in difficult situations, which was especially helpful for finding quick solutions. Good team spirit and sense of community were also underlined by most of the workers. It was stated that colleagues cared for each other and that an open question culture was promoted:

*“Well, advice from colleagues, that goes really fast. We simply go from door to door or we arrange to meet, that goes pretty well and works out pretty well.” [#8, male, homeless aid].*

Moreover, supervisors’ support was mentioned. For example, it was specified that they were approachable for work-related questions and particularly supportive in difficult situations at work:

*“And that we also have a boss who is receptive to us and takes us seriously. That’s also worth a lot and I have had completely different experiences with that.” [#2, female, homeless aid].*

#### Team meetings

Team meetings were also pointed out as an important source of support by several workers. In many cases, meetings in small teams were held once a week. Meetings in larger teams, e.g. cross-departmental or cross-location meetings, took place every 2 weeks, once a month or every 3 months. Team meetings were predominantly regarded as helpful for regular exchange and, in some cases, for collegial case consulting. However, some participants perceived the meetings as too superficial or too short to provide enough time for detailed discussion:

*“Case assessments are sometimes too short because in team meetings we have to talk about organisational issues and things like that, and in the end case discussions are neglected a bit.” [#20, female, homeless aid].*

Team meetings were sporadically used to develop concrete measures for improving the work conditions. Examples for such measures were the provision of number assignments for clients for open consultation hours, the use of stop signs indicating that there was no consultation hour, and the provision of mobile phones for communication with clients. Three workers also explained that conceptual and strategic planning took place on so-called “concept days” in the institutions:

*“Well, we do concept days here where we get together and talk and think about next steps. And actually I find that pretty ideal here.” [#10, male, homeless aid].*

#### Supervision

Supervision was another important source of support for many interviewees. It was typically provided once a month and in the form of group supervision. Some participants described larger time periods for supervision, e.g. once every 6 or 8 weeks. Two workers indicated that group supervision was not available to them. For the majority of the workers, supervision was perceived as helpful for self-reflection, knowledge exchange and for learning new tools and work methods:

*“I find supervision helpful because then somebody external comes along and sometimes you’re sort of a bit out on ledge when you’re just doing things yourself. And that just gives another perspective, which is often simply a relief.” [#3, female, homeless aid].*

However, dissatisfaction with supervision was also occasionally expressed, e.g. that it had to be postponed or cancelled due to understaffing, or that group supervision was not welcomed by all team members. The availability of individual supervision varied: for most of the interviewees, this form of supervision was not available. One worker stated that individual supervision was generally available, another said that it was only available for managers, and two interviewees declared that it was only available in extremely worrying situations (e.g. stalking, sexual abuse).

#### Training courses

The provision of training courses was another frequently mentioned source of support. Courses covered a range of topics, e.g. legal aspects, de-escalation techniques, counselling know-how and management skills. In some cases, there was a training budget available for each employee, and employees could select and request training themselves:

*“I am offered [training], internally or sometimes I can even make suggestions and organise things myself. And taking part in training with other providers is also approved and financed. That’s important.” [#12, male, refugee aid].*

#### Support from other institutions

Single interviewees described further sources of support within their institutions, e.g. support provided by experts from the human resources department or the sponsoring association. With respect to support from external sources, three workers stated that there were no other institutions to which they could turn for advice. The other workers described several sources of support in the form of network centres and counselling services (e.g. for issues like violence, drugs, debt and flight). Furthermore, sources of support also included federal associations, lawyers, doctors, psychologists, training providers, former professors and colleagues, volunteers or professionals with similar tasks who could be asked for advice. Cooperation with other institutions was generally perceived as supportive, as it allowed the interviewees to pass clients on to other parties if they were not able to provide support in all necessary aspects:

*“A large part of the work is actually that we look to see where there are places where we can send our people if they need special or concrete help.” [#15, male, refugee aid].*

#### Support in private life

Employees also described sources of support in their private lives provided by partners, families, friends and roommates. Support was particularly noticed when people in the family or circle of friends had a refugee background themselves and could assist with translation or interpreting tasks. Conversations with family members were also generally perceived as supportive:

*“Well, in my family we talk about the topic of homelessness a lot (…). My partner is also very interested in what I do and asks about it, so does my family.” [#8, male, homeless aid].*

### Workplace health promotion

Comments on workplace health promotion concerned the availability of health promotion offers as well as the further needs and wishes of employees.

#### Available health promotion offers

Overall, six respondents declared that no health promotion offers were available at their workplace, although some of them indicated the possibility of available health promotion offers that were unknown to them, e.g. due to the size of their organisations:

*“Maybe it does exist. But I think I just don’t know anything about it. Around Germany, they [the institutions] have up to 14,000 workers, and (…) a lot of roles and facilities and it is sometimes not really clear and you don’t always find out about everything.” [#14, female, refugee aid].*

Other workers reported several health promotion activities which consisted of individual offers rather than systematic workplace health management. With regard to behavioural measures, eleven interviewees stated that health days were organised every year or every 2 years. Two workers stated that there were massage services offered. These were either cross-departmental health days for all employees or organised individually by smaller teams. Moreover, respondents mentioned that courses and workshops were offered on different topics, ranging from stress management, mindfulness, yoga and relaxation to back training, healthy cooking and acupuncture. Quick relaxation and sports exercises were also occasionally organised, e.g. in the form of active lunch breaks. Furthermore, one respondent said that there was a volleyball group and another stated that there was an intern who had given the team Kung Fu lessons.

With respect to structural measures, four interviewees reported that companies offered medical examinations, vaccinations and funding for glasses at work. In addition, five workers reported funding for the use of gyms and two for fruit purchases at work. The implementation of a risk assessment on mental stress, regular health and safety information, funding for participation in company runs, and a service bike offer were described by one worker each.

In terms of workers’ experiences with health promotion offers, some employees stated that they had already used the offers or planned on doing so in due course. Six workers who had not used any offer so far indicated various reasons for not having done so, e.g. a lack of interest in existing offers and lacking motivation after work. In addition, the preference to take part in sport offers privately rather than in the work context was expressed by seven employees:

*“I mean, doing exercise activities with my colleagues isn’t really my thing. Doing Thai Chi on the roof with people, I would find that a bit weird. Because I do my own exercise.” [#5, female, refugee aid].*

Two respondents cited shift work as a major obstacle to participation in health promotion activities. Moreover, excessive workload and a resulting lack of time were described. One worker said that courses took place at unfavourable times and two commented that they took place in unfavourable locations (e.g. offers held in headquarters, but not in branch offices). Furthermore, the unclear and time-consuming registration process was noticed. Three respondents said that they would have to initiate and organise health promotion activities themselves, since this was not organised by the institutions.

### *Needs and wishes for health promotion offers*

In terms of behavioural measures, several respondents stated their general interest in sports offers and in the organisation of company sports groups (e.g. a running group after work):

*“Doing some sort of exercise activities with colleagues, some communal activities. Just that we do something to release a bit of energy and have a laugh, laughing is important.” [#7, female, homeless aid].*

Requests for regular, company-wide training courses were also made. Such courses should consist of a mixture of theory and practice and deal with various topics, e.g. back therapy training, stress management, relaxation techniques, qigong, yoga, body awareness, de-escalation and self-care. Three respondents particularly wanted activities to be offered during their work hours. Moreover, easily accessible activities at different times of the day were preferred and considered necessary for coordination with shift work:

*“You would really have to be able to choose when you go. (…) That you really can go in the mornings, maybe in the evenings, because, you know, we do a lot of shift work.” [#23, male, homeless aid].*

Four workers reported their desire for massages at work and two wished for a massage chair. Regarding structural measures, financial support for private hobbies and discounts for nearby gyms were desired. Moreover, further supervision, contact to lawyers for legal questions and regular medical examinations were stated. In addition, one respondent introduced the idea of a social worker counsellor working in the organisation’s facilities (instead of hard-to-reach external supervision):

*“If someone were to come into the facility and simply offer an open space to talk, somewhere you can simply drop in. Someone that doesn’t work here themselves, but who you could go to and simply talk about things, especially during working hours. A social worker for social workers.” [#6, female, refugee aid].*

With regard to the work environment, separate rooms for exercise and relaxation activities as well as rest rooms for breaks were requested. Two workers indicated their satisfaction with the available health promotion offers and stated that they had no need for further offers.

## Discussion

To our knowledge, this is the first study to empirically explore the coping strategies, support sources and health promotion offers available to German social workers in the growing work areas of refugee and homeless aid. By conducting qualitative interviews with 26 social workers in these fields, we were able to gain important new insights into these topics and extend current evidence.

Some of the problem-focused coping strategies identified in this study have previously been reported for social workers, such as the strategies of setting limits and boundaries in contact with clients or with regard to work and private life [5, 16, 17]. Moreover, acquiring knowledge (e.g. through training activities) in order to manage work-related stress has also been stated before by German refugee aid workers [27]. In addition to this, our study uncovered further problem-oriented strategies that have received little attention so far, such as strategies related to employees’ work organisation and time management. Time management coping strategies were described as helping workers prioritise and make the best use of their time, which was particularly important in view of their multiple work tasks and restricted time resources.

Another important coping strategy revealed in our study was the search for social support. This strategy emerged both in the context of problem-oriented coping strategies (seeking instrumental support from colleagues and superiors to deal with stressful situations and concrete problems at work) and emotion-oriented coping strategies (seeking informal social support from family and friends to alleviate negative emotions). In general, the search for social support is a frequently used coping strategy, and protective links between social support and health are well documented [48, 49]. The finding is consistent with previous research in which social support represented an essential job resource for staff in refugee and homeless aid [6, 50].

In addition, a notable finding of our study is that some interviewees had started psychotherapy to better cope with their job demands and associated strains. The fact that psychotherapy was initiated by the interviewees themselves points to a high level of suffering among these workers, which was emphasized by the workers themselves. In accordance with this, previous studies have shown a high prevalence of long-term psychological strain reactions among social workers, including depressive moods and burnout [9, 13, 51, 52]. The result is somewhat alarming, as it suggests a perceived lack of possibilities for the workers to address their problems in the workplace and receive adequate help, e.g. through individual supervision. In fact, individual supervision was unavailable to most workers in our study. Earlier research suggests that the availability of individual supervision may vary across different settings and countries. For example, in a recent study on homeless aid in the UK, 83% of the frontline workers had access to individual supervision [26].

With respect to the reported emotion-oriented coping strategies, being active (e.g. exercising, taking walks outdoors) and pursuing leisure activities were underlined by many workers as central coping behaviours. The importance of these strategies has been outlined before [5, 6, 16, 17]. Moreover, actively organising their leisure time helped the workers to get away from work. Evidence suggests that social workers often find it difficult to switch off from work [13, 17], which further explains their use of coping strategies that enable them to detach and recover from job stress.

Further emotion-focused coping strategies employed by the interviewees were the strategy of avoiding presenteeism and consciously taking self-care actions. Similarly, the use of self-care strategies (e.g. diet changes, improvement in sleep hygiene, relaxation) was found to be a functional coping strategy [21] which was also used by employees in homeless aid [6].

Notably, several emotion-oriented coping strategies used by the workers in our study involved cognitive components, e.g. gaining mental distance from work, accepting unchangeable situations and focusing on positive experiences. In earlier studies with social workers, similar cognitive coping efforts were described, e.g. with regard to the acceptance of clients’ undesirable behaviour without taking it personally [19] and of their boundaries of influence [18]. The use of cognitive coping strategies may be particularly efficacious for social workers when they encounter problems that cannot be changed directly (e.g. political laws). However, making use of such strategies is also demanding, as it firstly requires the workers’ ability to reflect problems on a meta level.

In earlier research, most of the coping strategies employed by social workers were classified as emotion-focused [9]. In comparison, employees in our study used both problem-oriented and emotion-oriented strategies, although a slight tendency towards the use of more emotion-oriented strategies was observable. Referring back to the theoretical definition of coping provided by Lazarus and Folkman [36, 37], the variety of identified coping strategies underpins the notion that most individuals use both problem-oriented and emotion-oriented coping strategies to deal with stressful events [36].

Regarding the identified sources of support for social workers, most of them were linked to workplace social support [40], e.g. provided by team members, supervisors, external persons and institutions. This result underlines the importance of social interaction for the working group. It also suggests that the workers may already have some sources of support available in the work setting. At the same time, however, our study sheds light on potential for improvement, e.g. with regard to team meetings and supervision being postponed, too short or too superficial to provide help for complex problems. Our results are consistent with earlier research indicating that social workers (especially those with severe strain reactions) did not feel sufficiently supported by supervisors [13]. External supervision and consulting are important tools for reflecting on one’s work methods and stressors, and have shown protective effects on the health [53] and job satisfaction [25] of social workers. Conversely, a recent qualitative study suggested that inadequate supervision and a lack of supervisor support may play a critical role in the development of long-term strain among social workers [13]. Summarising the above, the need for adequate supervision for social workers in homeless and refugee aid has been raised before [9, 13] and is strongly reinforced by our results.

With regard to workplace health promotion, a relevant finding of our study is that several employees were unaware of health promotion offers at their workplaces, although some of them indicated that such offers could possibly exist. Furthermore, several workers named individual health promotion offers, but none of them described a systematic workplace health management. On the one hand, this suggests that there may be little systematic approaches to workplace health management within the organisations so far. On the other hand, the findings may also point to a lack of information on the part of the interviewees, suggesting that health promotion offers may not be advertised properly and that communication flows within the organisations need to be improved. Many of the cited needs for behavioural and structural health promotion measures are consistent with the results from earlier research. For example, in terms of behavioural measures, the respondents wished for regular and company-wide training courses on a range of topics, which has been similarly revealed in previous studies [16, 50]. A recent study showed that over one fifth of frontline workers in homeless services never had access to relevant training, e.g. relating to suicide, self-harm and mental health [26]. Some training options were especially scarce, e.g. training on trauma and domestic violence, which one third of the workers had never received. Needs in terms of structural measures concerned offers for supervision and counselling as well as changes in the work environment. Likewise, in a recent study, it was demanded that employees should be provided with adequate facilities to enable relaxation during their breaks [13].

### Implications

From the results of our study, some implications for research can be drawn. In terms of research-related implications, quantitative studies with higher sample sizes should be carried out to generalise and quantify our findings, e.g. regarding the potentially positive effects of coping and the status quo of workplace health promotion for social workers in refugee and homeless aid. In future studies, it would be interesting to compare the support sources and health promotion offers for social workers in different work settings (e.g. organisations of different types and sizes, with independent and public sponsors, and in rural and urban structures). This could help to gain a deeper understanding of the beneficial and impeding factors for implementing health promotion offers.

Practical implications can also be derived (Table 5). Our findings indicate that workplace interventions should be carried out aiming at empowering social workers to further expand upon their coping strategies. In organised courses and workshops, employees could acquire relevant skills and learn about further ways of coping with job stress in a resilient manner. With respect to the development of support sources, demands for greater support and expanded supervision for social workers have previously been made [53] and are strongly reinforced by our findings. Supervision is highly valuable, as it promotes reflection, support and evidence-based expertise [53]. To support employees in dealing with their emotional demands, easy and low-threshold access to qualified supervision must be provided for all workers in the form of individual and/or group supervision. This seems particularly relevant in view of the stress factors and secondary/post-traumatic stress in social workers serving refugees and homeless clients [14, 15]. In the same vein, it is important to inform the workers well about the usefulness of supervision, especially those who are still inexperienced and unsure about using this offer. Moreover, team support within the institutions should be nurtured and upheld. For this purpose, the provision of regular meetings with sufficient time for case consulting and enough room to spend breaks together with colleagues would be useful. Emphasis should also be placed on networking, regular exchange and cooperation with other counselling services which can provide support as neutral entities.

**Table 5:** Study topics and related practical implications

Topic	Aim	Examples for practical implications
Coping	Empowering social workers to expand effective coping strategies	Organised courses and workshops to acquire relevant skills and learn about ways of coping with stress in a resilient manner
Sources of support	Increasing support sources for social workers within the institutions	Easy and low-threshold access to qualified supervision; regular team meetings with case consulting, opportunities for networking, exchange and cooperation with counselling services
Health promotion measures	Implementing structural and behavioural measures to foster social workers’ health	Structural measures: e.g. to improve the work organisation (reliable work hours, manageable workload) and environment (more rooms for rest and recovery). Behavioural measures: e.g. training courses on work- and health-related topics (exercise, relaxation, de-escalation)

In view of the diverse needs for health promotion stated by the workers, our findings provide a useful starting point for planning needs-based health promotion offers. Suitable structural measures may address the work organisation (e.g. in terms of ensuring reliable work hours and a manageable workload). They should also aim at improving the work environment, e.g. by providing more rooms for rest and recovery. With respect to behavioural measures, training courses on work- and health-related topics (exercise, relaxation, de-escalation, violence, etc.) are recommended. For example, since workers in our study mentioned aggressive behaviour of clients, the availability of training courses on de-escalation and non-violent communication may be particularly helpful for those who encounter such problems at work. Our results indicate that initial health promotion offers are already available at many organisations, meaning that future interventions can be based on existing offers and can expand upon them, taking the workers’ needs into account. Employees should be given opportunities to make flexible use of health promotion offers, e.g. during work hours and at different locations in order to improve coordination with shift work, which is often a major obstacle in participation. Since health promotion offers may not always be well-promoted, this highlights the importance of systematic and target-oriented advertisement within the organisations. After all, politicians at a higher level also have a responsibility for creating a framework and providing sufficient resources to promote these crucial fields of social work, so that adequate programs can be implemented at company level.

### Strengths and limitations

A particular strength of this study is that the views of social workers were assessed in a heterogeneous sample (e.g. in terms of age, work experience, etc.). Thereby, we were able to capture different perspectives and broadly map the topics of interest. Considering the explorative character of the study, a sufficient number of workers was included in order to attain data saturation [54]. Further strengths of this study are the consistent orientation towards and application of recognized field practices, e.g. the data analysis according to Mayring’s qualitative content analysis [44]. To improve the internal quality of the study, we used rich descriptions as well as numerous direct quotes to describe our results [55], and applied the international checklist “COREQ” [47]. Moreover, all results were compared to empirical references and to the theoretical framework [56].

Some limitations of the study should also be noted. As in any qualitative study, the interviewees’ responses may have been influenced by the lack of anonymity between the workers and the interviewer as well as by social desirability tendencies. Selection effects cannot be ruled out either. For example, all respondents spoke fluent German and were rather young; results could be different for older employees or employees of other nationalities. Qualitative research captures the subjective perspectives and truths of the respondents. The interview sample is not representative of the general population of social workers, and the results are not generalisable to other settings or time periods. The temporal context of the study period should also be noted: the interviews were conducted in autumn 2017, when immigration in Germany slowly subsided, leading to restructuring measures and closures of institutions. In homeless aid, employees experienced a steady increase in homelessness and changes in the clientele in terms of, for example, gender and origin [57]. Such recent developments should generally be kept in mind with regard to their potential impact on our results.

## Conclusions

The results of the study provide novel insights into the coping strategies employed by social workers in refugee and homeless aid and into the available support sources and workplace health promotion. On the one hand, the findings show that social workers use multiple coping strategies and have access to different support sources in the workplace, helping them to deal with their job demands and regulate their emotional responses. On the other hand, the results suggest that certain needs for support among employees are not yet covered, and that systematic workplace health promotion appears to be scarce within the organisations. The identified wishes of the workers for behavioural and structural measures are particularly relevant for health promotion, as they indicate diverse areas and starting points for policy makers and organisations to design needs-based health promotion interventions for social workers in refugee and homeles aid.

## Supplementary information

**Additional file 1.** Relevant interview guideline questions.

**Additional file 2.** COREQ-checklist.

## Data Availability

The datasets are not publicly available due to German national data protection regulations. They are available from the corresponding author upon reasonable request.
