# Supplementary material for: “I need to take care of myself”: a qualitative study on coping strategies, support and health promotion for social workers serving refugees and homeless individuals
Source: J Occup Med Toxicol. 2020 Jun 26;15:19. doi: 10.1186/s12995-020-00270-3 (PMC7318459; doi:10.1186/s12995-020-00270-3)
Supplement: Supplementary file 1 — Additional file 1. Relevant interview guideline questions. [file 12995_2020_270_MOESM1_ESM.pdf]

Additional file 1. Relevant interview guideline questions

Interview topic	Interview questions
**Coping strategies at work and in private life**	(1) How do you deal with stressful situations at work?<br>(2) What do you do in your free time to recover and mentally detach from work?
**Sources of support at work and in private life**	(1) Do you receive support at your workplace if you have technical questions or need collegial advice? If so, from whom?<br>a. Do you have regular team meetings and/or meetings for supervision at your workplace? Are these helpful?<br>(2) In addition, are there any further institutions or individuals you may contact if you need work-related advice?<br>(3) What further help or support would you wish for your work?<br>(4) Do you also receive private support for work-related problems?
**Health promotion**	(1) Are health promotion offers provided to employees at your workplace? If yes:<br>a. What options are available?<br>b. Who provides them?<br>c. Do you make use of these offers?<br>d. What experiences have you had with health promotion?<br>e. Would you like any additional options to be offered?<br>If not: Would you like health promotion to be offered at your workplace? What options would you like to see?
