# Supplementary material for: “I need to take care of myself”: a qualitative study on coping strategies, support and health promotion for social workers serving refugees and homeless individuals
Source: J Occup Med Toxicol. 2020 Jun 26;15:19. doi: 10.1186/s12995-020-00270-3 (PMC7318459; doi:10.1186/s12995-020-00270-3)
Supplement: Supplementary file 2 — Additional file 2. COREQ-checklist. [file 12995_2020_270_MOESM2_ESM.pdf]

Supplementary Table 1. Consolidated criteria for reporting qualitative studies (COREQ): 32-item checklist

A checklist of items that should be included in reports of qualitative research.

Tong A, Sainsbury P, Craig J. Consolidated criteria for reporting qualitative research (COREQ): a 32-item checklist for interviews and focus groups. International Journal for Quality in Health Care. 2007. Volume 19, Number 6: pp. 349 – 357

Topic and Item No.	Guide Questions/Description	Reported on Page No.
**Domain 1: research team and reflexivity**		
Personal Characteristics		
1. Interviewer/facilitator	Which author/s conducted the interview or focus group?	8
2. Credentials	What were the researcher's credentials? E.g. PhD, MD	8
3. Occupation	What was their occupation at the time of the study?	8
4. Gender	Was the researcher male or female?	8
5. Experience and training	What experience or training did the researcher have?	8
Relationship with participants		
6. Relationship established	Was a relationship established prior to study commencement?	8
7. Participant knowledge of the interviewer	What did the participants know about the researcher? *e.g. personal goals, reasons for doing the research*	8
8. Interviewer characteristics	What characteristics were reported about the interviewer/facilitator? e.g. *Bias, assumptions, reasons and interests in the research topic*	8
**Domain 2: study design**		
Theoretical framework		
9. Methodological orientation and Theory	What methodological orientation was stated to underpin the study? *e.g. grounded theory, discourse analysis, ethnography, phenomenology, content analysis*	7
Participant selection		
10. Sampling	How were participants selected? *e.g. purposive, convenience, consecutive, snowball*	7
11. Method of approach	How were participants approached? *e.g. face-to-face, telephone, mail, email*	7
12. Sample size	How many participants were in the study?	7
13. Non-participation	How many people refused to participate or dropped out? Reasons?	7

Setting		
14. Setting of data collection	Where was the data collected? *e.g. home, clinic, workplace*	7
15. Presence of non-participants	Was anyone else present besides the participants and researchers?	7
16. Description of sample	What are the important characteristics of the sample? *e.g. demographic data, date*	9-10
Data collection		
17. Interview guide	Were questions, prompts, guides provided by the authors? Was it pilot tested?	8
18. Repeat interviews	Were repeat interviews carried out? If yes, how many?	8
19. Audio/visual recording	Did the research use audio or visual recording to collect the data?	8
20. Field notes	Were field notes made during and/or after the interview or focus group?	8
21. Duration	What was the duration of the interviews or focus group?	8
22. Data saturation	Was data saturation discussed?	8
23. Transcripts returned	Were transcripts returned to participants for comment and/or correction?	8
**Domain 3: analysis and findings**		
Data analysis		
24. Number of data coders	How many data coders coded the data?	8-9
25. Description of the coding tree	Did authors provide a description of the coding tree?	8-9
26. Derivation of themes	Were themes identified in advance or derived from the data?	8-9
27. Software	What software, if applicable, was used to manage the data?	8-9
28. Participant checking	Did participants provide feedback on the findings?	9
Reporting		
29. Quotations presented	Were participant quotations presented to illustrate the themes / findings? Was each quotation identified? *e.g. participant number*	11-20
30. Data and findings consistent	Was there consistency between the data presented and the findings?	11-20
31. Clarity of major themes	Were major themes clearly presented in the findings?	11-20
32. Clarity of minor themes	Is there a description of diverse cases or discussion of minor themes?	11-20
